# Clinical and Forensic Aspects of the Different Subtypes of Argyria

**DOI:** 10.3390/jcm10102086

**Published:** 2021-05-13

**Authors:** Luís Mota, Ricardo Jorge Dinis-Oliveira

**Affiliations:** 1 Department of Public Health and Forensic Sciences, and Medical Education, Faculty of Medicine, University of Porto, 4200-319 Porto, Portugal; 2 IINFACTS-Institute of Research and Advanced Training in Health Sciences and Technologies, Department of Sciences, University Institute of Health Sciences (IUCS), Advanced Polytechnic and University Cooperative (CESPU), CRL, 4585-116 Gandra, Portugal; 3 UCIBIO-REQUIMTE-Applied Molecular Biosciences Unit, Laboratory of Toxicology, Department of Biological Sciences, Faculty of Pharmacy, University of Porto, 4050-313 Porto, Portugal

**Keywords:** argyria, pathophysiology, signs and symptoms, clinical and forensic diagnosis, treatment

## Abstract

Argyria encompasses the different cosmetic alterations that can develop if enough silver particles deposit in a specific tissue, typically in the skin, ranging from localized dark-blue macules to a generalized slate-gray/bluish tinge following systemic absorption. This work aims to fully review the state of the art regarding pathophysiology, diagnosis, treatment, and relevant clinical and forensic features of argyria. Argyria has been diagnosed in a wide range of ages, both sexes and varied ethnicities, with no known individual predisposing factors. Ultraviolet radiation with subsequence increases of melanin production aggravates the discoloration due to a reduction in the silver deposits. Physical examination and silver exposure in the anamnesis can be highly suggestive of the diagnosis, but a histopathological analysis with Energy-Dispersive X-ray Spectroscopy is required to unequivocally determine the discoloration etiology. Safe and effective treatment has only been accomplished with laser techniques, though only a few cases have been reported and with limited follow-up time. In conclusion, argyria typically has an occupational or iatrogenic etiology. It should be suspected when a patient presents with typical skin or eye lesions. A seemingly viable treatment modality, with laser technology, is finally within the horizon.

## 1. Introduction

Argyria refers to inert silver deposition in a tissue, typically the skin, resulting in characteristic blue/gray spots or a diffuse hue, but also possibly darker or brownish [1,2,3,4,5,6]. It occurs following excessive accidental voluntary cumulative silver exposure by the most varied causes. It is mainly a cosmetic condition whose clinical presentation will vary according to the subtype as describe below [2,3,7,8,9].

The state of the art concerning pathophysiology, clinical presentation, diagnosis, therapeutic modalities, and forensic features of argyria is reviewed, and the main gaps in current knowledge, where future research ought to be focused, are highlighted.

## 2. Materials and Methods

An exhaustive search was carried out in PubMed database without a limiting period concerning pathophysiology, signs and symptoms, history and physical examination, diagnostic, treatment, and forensic aspects of argyria. The keyword “argyria” was searched in articles written in all languages. Additionally, the keyword was crossed with diagnosis, toxicokinetics, amalgam tattoo, ocular argyrosis, azure lunula, and treatment. Furthermore, retrieved journal articles, as well as books and governmental documents, were also reviewed for possible additional publications related to this topic. A total of 290 scientific documents, including books, articles, and government documents, were considered for this review.

## 3. Chemistry and History of Silver

Silver derives from the Anglo-Saxon “seofor” and “siolfur”, and it is a chemical element, solid at room temperature, classified as a transition metal (atomic number 47), of period 5 and group 11 of the Periodic Table. Its symbol is Ag, which derives from its Latin name *argentum* [10,11]. It is a rare, naturally occurring element found as a soft, “silver”-colored metal in its pure form, but in the environment, it is mostly found in its typical oxidation state (+1) combined with other ions or molecules, such as sulfide, chloride, and nitrate, which give the compound a color ranging from dark-gray/black to powdery white [11,12]. Of all known metals, silver has the highest electrical and thermal conductivity and possesses the lowest contact resistance [11].

Silver value has been recognized since ancient times, as it was likely separated from lead as early as 4000 BC [10]. Silver metal is used for jewelry, silverware, electronic equipment, and dental filling. Soluble inorganic silver salts (e.g., silver nitrate and silver sulfide) are strongly bactericidal, and particularly silver nitrate (1%) had been used immediately after born to prevent gonococcal ophthalmitis in newborns. Antibiotic ointments have replaced silver nitrate for this indication. Silver sulfadiazine slowly releases silver and is used to suppress bacterial growth in patients with second- and third-degree burns wounds. Nevertheless, silver medicinal value is often overemphasized, and unsubstantiated claims for treating diverse diseases are widely reported. Occupational exposure occurs mainly from inhalation of silver fumes and dust in different settings; for instance, silver halide is used in the manufacture of photographic plates [13,14].

There is no essentiality for silver, and dietary intake is in the range of 0.4 to 27 μg/day, which is much less than the silver intake from medicinal uses [15]. For drinking water disinfection, World Health Organization permissible level is reported to be 0.1 mg Ag/L [16]. One of the recent applications of silver is in nanoparticles (AgNP), materials with sizes ranging from 1 to 100 nm, containing 20 to 15,000 atoms of silver. Because of their antimicrobial activity, AgNP are used in a variety of consumer products, including medical devices, disinfectants, appliances, textiles, and water treatment [15,17].

## 4. Toxicokinetics of Silver

Silver is primarily absorbed by the gastrointestinal tract, lung, and skin [15,16,18]. Up to 10% of ingested silver, a value expected to vary with individual characteristics (at least age, health, and nutritional status) and the degree of ionization/solubility of the silver compound, is absorbed in the gastrointestinal tract [3,13,19,20,21,22]. Metallic silver and insoluble silver compounds are not readily absorbed and pose a minimal health risk, unlike particulate or colloidal silver, whose toxicity comes from released ionic particles [13,15]. Inhalation of aerosolized particles, typically occurring in the occupational setting, is also a relevant pathway for silver absorption, but the toxicokinetic properties of this process remain even more uncharacterized [21,23,24,25,26]. There has been some consensus around 0.01 mg/m^3^ as the threshold limit value for daily silver occupational exposure, but a lower limit of 0.1 mg/m^3^ for metallic silver is becoming more common, as it shows less propensity to cause argyria than its ionic, more soluble and absorbable counterpart [27].

Silver is transported in the bloodstream as a colloid, in its ionic form (Ag^+^), stabilized by complexing with proteins, mainly albumin but also globulins [20,24,28,29]. It is widely distributed to most tissues, such as muscle, cerebellum, spleen, duodenum, heart, lung, liver, and kidney [15]. It is not clear whether silver crosses the blood-brain barrier, although several studies indicate accumulation in specific areas of the brain [15]. Indeed, silver deposition has been documented in the blood-brain barrier (vascular endothelium and astrocytes), the blood-cerebrospinal fluid barrier (choroid plexus), and the cerebrospinal fluid itself [30,31,32]. Moreover, Landas et al. [33] reported a *postmortem* evaluation with silver deposition in circumventricular organs and hypothalamic nuclei, suggesting silver’s eventual passage to the central nervous system. Nevertheless, the state of the art is far from being clarified.

The primary routes of exposure for AgNP include excretion occurring predominantly in the bile but also through the urinary system in a much lesser amount [3,19,20,28,34,35,36]. Indeed, biliary excretion is important in the homeostasis of several metals, notably copper, manganese, cadmium, selenium, gold, silver, and arsenic [37].

## 5. Argyria Subtypes

Silver deposition has been documented in several anatomic places such as the skin, eyes, kidneys, and liver, but it might happen in virtually every organ, which is coherent with silver’s general affinity for stromal tissue and basement membranes and, as in the skin, this deposition has not been definitely associated with any harmful effects [20,38,39,40,41,42,43]. In the following sections, different types of argyria are reviewed.

### 5.1. Generalized Argyria

**Figure 1:**
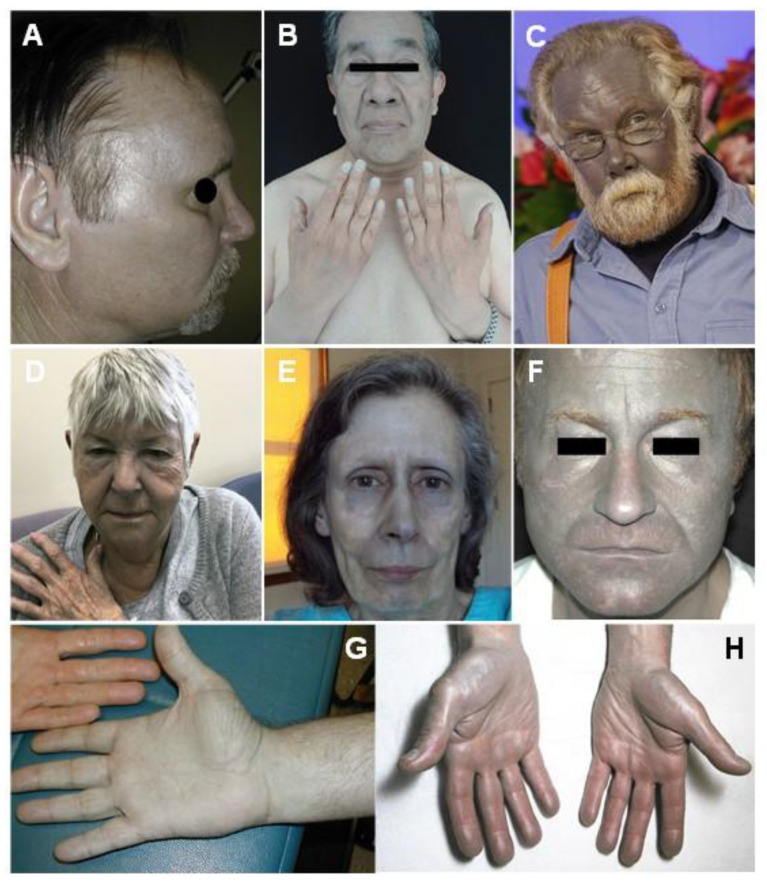
Characteristic bluish-grayish signs of generalized argyria, namely in sun-exposed areas of the head, neck, and hands. Reprinted from (**A**,**G**)—[20], (**B**)—[58], (**C**)—(Today media news), (**D**)—[34], (**E**)—[59], (**F**)—[47] and (**H**)—[60].

Generalized argyria (GA; Figure 1) emerges following silver systemic exposure and its uptake by the dermis [3,5,8,24,44,45]. This leads to a gray/blue saltish or metallic diffuse skin pigmentation, which becomes evident predominantly in sun-exposed areas [3,5,38,44,46,47,48]. Patients were most commonly present with gradually aggravating face and neck discoloration with a history of oral but also occupational aerosolized exposure to silver-containing products [3,13,28,44,49,50,51,52,53,54]. Different minimal amounts of elemental silver cumulative oral intake able to produce GA have been suggested, ranging from 2 to 30 g, but such values provide incomplete information unpaired with a time window [8,19,29,39,55,56,57].

**Figure 2:**
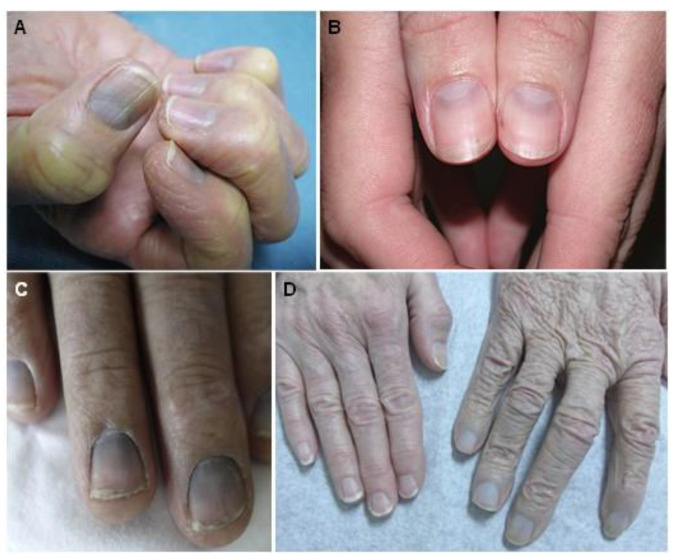
Characteristic bluish-gray discoloration of the nails (azure lunula), namely of the lunula due to silver exposure. In D comparison with regular nails is provided. Reprinted from (**A**)—[67], (**B**)—[68], (**C**)—[66], and (**D**)—[69].

Azure lunula is a bluish discoloration of the fingernails, more precisely of the lunula (Figure 2), but with the possible extent to the proximal half of the fingernail that was frequently accompanying GA [6,36,47,50,61,62]. Another possible early sign of GA is acquired pigmentation of the oral mucosa; unlike amalgam tattoos, a diffuse gray/blue tinge will be seen [1,6,7,49,58,63,64,65,66].

### 5.2. Localized Argyria

**Figure 3:**
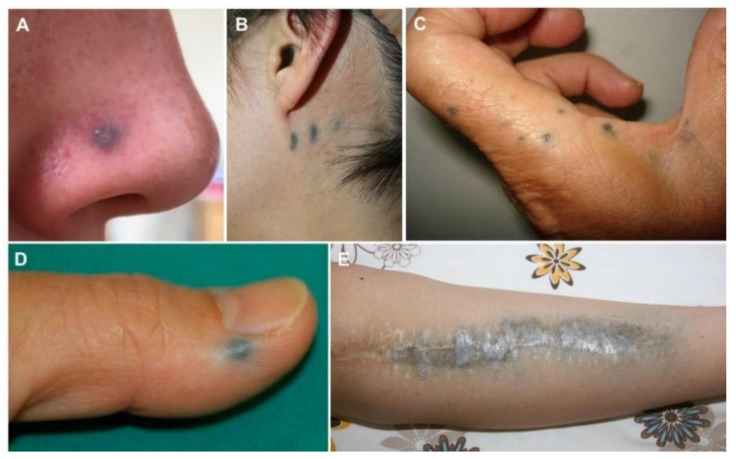
Characteristic dark-bluish-grayish macules of localized argyria following a silver jewelry nose piercing (**A**), acupuncture treatment (**B**), silver jewelry in the index finger (**C**), and macule-mimicking a nevus (**D**). Discoloration of the leg over a silver-coated megaprosthesis used for distal femoral osteosarcoma (**E**). Reprinted from (**A**)—[88], (**B**)—[89], (**C**)—[87], (**D**)—[73] and (**E**)—[90].

Localized argyria (LA; Figure 3) is less commonly seen, being caused by local silver deposition following skin incisions or percutaneous absorption via sweat gland pores [3,8,28,70,71,72,73,74,75,76,77,78,79,80]. This results in macular lesions or spots-clusters, confined to where the silver impregnation occurred and with coherent morphology, whose color, compared to GA’s, tends to be darker, sometimes almost black [8,73,81,82,83]. Patients most commonly present with complaints of asymptomatic lesions in a site of previous trauma, cautery with silver devices, or prolonged contact with silver-containing objects, creams, or solutions, as it occurs in the hands and forearms of silver-handling workers [2,75,81,82,84,85,86,87].

**Figure 4:**
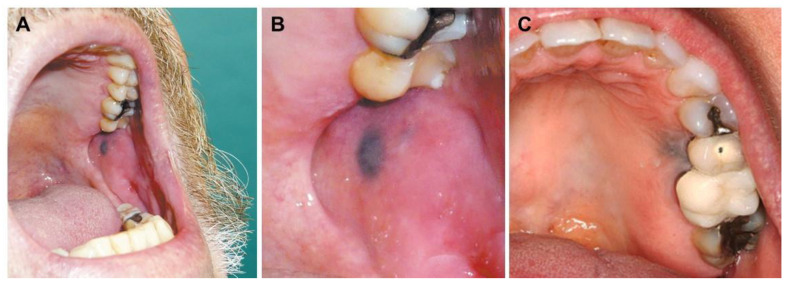
Amalgam tattoo characterized by the dark-blue macule on the buccal mucosa near a restored tooth. Reprinted from (**A**,**B**)—[95], (**C**)—[110].

Amalgam tattoo (Figure 4) is probably the most common form of LA [91,92,93]. It is is a common and easily recognizable entity that results from the impregnation of silver-contained dental amalgam into oral mucosa following tooth restoration procedures [92,94,95,96,97,98]. A flat, dark-blue mucosal lesion near a restored tooth is highly indicative of an amalgam tattoo, with size varying from small, millimetric lesions to larger ones, with a minority of these even exhibiting radiopacity and potentially triggering a foreign body reaction [92,95]. Besides silver, it is important to highlight that dental amalgams could also present silver, tin, copper, and zinc, which may also have a role in the development of amalgam tattoos [99]. A biopsy might be useful, especially to rule out melanoma [92,95,96,100]. Other less common forms of LA reported are in the nasal [101,102], tracheal and bronchial mucosa [103], urinary tract [104,105,106,107], vagina [108], and penis [109].

### 5.3. Argyrosis

**Figure 5:**
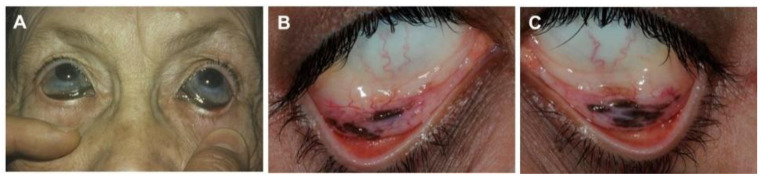
Ocular argyrosis characterized by intense dark pigmentation of the palpebral conjunctiva, with a more discrete bluish hue of the bulbar conjunctive (**A**) and of the palpebral conjunctiva (**B**,**C**), mimicking a conjunctival melanoma. Reprinted from (**A**)—[123], (**B**,**C**)—[119].

Argyrosis is a particular argyric manifestation evidenced by the ocular silver deposition (Figure 5) that can occur in GA [6,34,47,49,54,111,112], but also as a LA form [113,114,115,116]. It is mostly detected in the cornea, bulbar and palpebral conjunctivae, and lacrimal caruncle [113,114,117,118,119,120]. Its appearance ranges from small, darker lesions to a more disperse tinge, somewhat parallelly to LA and GA, and with greater heterogeneity concerning coloration, as greenish and brownish tones might be displayed other than the typical gray/blue [121,122].

## 6. Pathophysiology of Argyria

Covalent adduct formation is common with electrophilic compounds because they react with nucleophilic atoms in proteins and nucleic acids. In general, soft electrophiles prefer to react with soft nucleophiles (low charge-to-radius ratio in both), whereas strong electrophiles react more readily with strong nucleophiles (high charge-to-radius ratio in both), such as the oxygen in nucleic acids. Metal ions as silver and mercury are soft electrophiles that react covalently with soft nucleophiles, particularly thiol groups, such as cysteine-abundant collagen fibers and proteoglycans in the extracellular matrix [3,29], and metallothioneins intracellularly, whose synthesis is induced after cellular silver intake [24,30,77,124].

Discoloration develops following ultraviolet exposure since silver ions undergo photoreduction to atomic silver, which can be oxidized mainly to low-solubility and chemically stable compounds such as silver sulfide (Ag_2_S) and silver selenide (Ag_2_Se) [13,29,125,126,127,128]. This photoreduction process is equivalent to the darkening of photographic film following light exposure and leads to argyria and explains its greater notoriety in sun-exposed areas [9,19,79,129,130]. In the absence of light as occurs in the gastrointestinal tract, argyric discoloration may be due to the role of tissue enzymes or other redox systems in the conversion of silver to its elemental form [40,112,130,131]. The subsequent formation of very low-solubility complexes can be compared to photographic toning/metal tarnishing, as it increases the stability of the silver aggregates and influences the color of the acquired pigmentation, and it also restraints even further silver’s interaction potential with biological structures, providing some explanation to its lack of toxicological effects and foreign body reaction generation [9,98,127,128].

There is evidence of silver found intracellularly, lying in the cytoplasm or bound to lysosome-like structures inside histiocytes and fibroblasts, suggesting a cellular uptake role in its cleanup, but not in any way near an extent that enables pigmentation resolution; therefore, silver is clearly found mainly in extracellular locations [3,29,80,81,128,132,133,134,135,136].

After silver gains access to the dermis or other tissues, either carried by the bloodstream (leading to GA) or localized (leading to LA), it deposits in a predictable pattern, as shown by histopathological and ultrastructural studies [51,70,76,81,85,137]. Typically, the silver particles settle in the connective tissue underlying epithelial surfaces, arranged in rows of granules, with a propensity for the basement membranes of blood vessels, eccrine sweat glands, and other dermal adnexa, but also depositing along dermal elastic fibers and the dermo-epidermal junction, while staying clear of the epidermis [4,7,55,64,73,79,81,85,133,134,138,139,140,141]. Of note, the outermost skin layer also influences the macroscopical appearance of the discoloration; since it reflects light in the violet/blue spectrum more than in longer wavelengths, argyria colors are usually perceived as blue/gray at clinical evaluation. In anatomic places where *stratum corneum* is thicker, color may be less intense; indeed, argyric patients’ hands, palms appear less pigmented than the dorsa, despite having a similar concentration of silver particles in the dermal tissue [45,71,77,79,142,143,144,145].

Also playing a role in skin darkening is silver’s stimulatory effect on melanin production, which appears to be one of the few direct effects of silver particles in surrounding tissues, possibly via an increment on tyrosinase enzyme activity [9,77,78,84,125,146,147].

Regarding azure lunula, the pathophysiology explaining the prevalence of discoloration in the lunula in comparison to nail beds is not yet clarified. What is known is that the nail plate is translucent, allowing the visualization of the underlying nail bed. It also grows thicker distally, being thinner in the lunula. With nail formation, cells move forward from the lunula (keratogenous zone), suffering fragmentation until they are predominantly anucleated, eosinophilic, and arranged in very compact sheets [148]. These two factors (nail plate thickness variation and changes in keratinocytes along the nail plate) might play a role in the color disposition found in argyric nails (azure lunula), as well as a richer vascularization toward the nail matrix when compared to the nail midbed [148].

In the eye, silver deposits exhibit a clear predilection for corneal Descemet’s membrane, but it can also be found in Bowman’s membrane, the stromal tissue in-between, both bulbar and palpebral conjunctiva and the lacrimal caruncle [25,58,102,113,149,150,151,152]. When the lacrimal sac is visualized (following dacryocystectomy), it often displays the pigmentation as well and in a striking fashion, with a strong black tone [114,153,154]. Reports of crystalline lens silver deposits have also been made [54,114,149,155]. In the posterior segment of the eye, silver accumulation is speculated about, following reports of retinal pigment epithelium changes, drusenoid deposits, and dark choroids in fluorescein angiogram, but a necessary causal relationship was not yet clearly established [111,156,157,158]. Following local contact, silver episcleral and periocular inoculation, such as in the eyelids, can also be present [23,86,159,160,161]. Silver’s histopathological pattern of deposition will also be similar whether there’s local implantation or systemic absorption, behaving as seen elsewhere in the body: settling in epithelial basement membranes and subepithelial connective tissue, predominantly extracellularly, and undergoing redox reactions that aggravate the pigmentation and stabilize the silver compounds [102,112]. Following systemic exposure, ocular argyrosis, more specifically corneal and/or conjunctival involvement, is seemingly the earliest indicator of silver accumulation in the body, with its deposition in these tissues being often documented before any other clinical signs of argyria [120,162]. Some authors have even claimed that there is a correlation between the number of eye deposits/degree of coloration and the length of silver exposure [122,163].

## 7. Diagnosis of Argyria

Argyria is often overlooked as a differential diagnosis of pigmented lesions, given its rarity [8,45,83,164]. Any of these findings might correspond to argyria: a slate-gray/bluish change of the complexion in sun-exposed areas, localized dark-blue lesions, eye gray/brown/dark spots or conjunctival pigmentation, or the characteristic nail changes seen in azure lunula [34,46,48,61,73,82,115,165]. Silver product exposition, as in oral silver consumption in a GA-suitable patient, a previous dental restoration in a patient presenting with amalgam tattoos, or acupuncture history in a patient with dispersing cutaneous LA lesions, can be highly indicative of argyria [5,53,86,89]. Different causes have been documented, and Table 1 compiles all major causes of argyria.

The timespan for the onset of the pigmentation might vary from days to several years, and it then becomes ever lasting, making the diagnosis of argyria not discardable regardless of the amount of time passed since exposition and first-time pigmentation observation [8,81,83,87,134,164,165,188,222,244]. These should prompt a thorough anamnesis to ascertain if there is any kind of pathological or toxicological justification for these alterations and to establish a differential diagnosis (Table 2).

### 7.1. Signs and Symptoms Related to Argyria

Signs and symptoms derived from silver exposure other than the cosmetic changes are usually absent, even after systemic absorption, suggesting that silver has low toxicity in humans [3,6,9,13,58,131,132,147,261]. The silver deposits in the skin do not seem to cause any harm to the surrounding tissues, being the psychosocial embarrassment derived from the aesthetic impact usually the most negative consequence, sometimes even leading to social withdrawal [9,43,47,55,132,223].

Patients with OA have reported visual symptoms, most commonly nyctalopia [54], and presented with concomitant comorbidities, such as glaucoma [151,163], cataract [114,149,218,262], diabetic retinopathy [111], as well as posterior eye segment changes somewhat coherent with the functional complaints [157,158], but without direct link to the silver deposition itself [156,162,263]. This inconsistency, alongside several OA patients lacking any visual complaints, has contributed to some consensus regarding the seemingly innocuous silver deposition in the eye, though this claim still needs a more robust foundation [21,113,261,263,264].

As silver has been found to settle in several tissues, concern has been raised regarding possible systemic toxic effects. Regarding the kidney, a decrease in the glomerular filtration rate has been suggested, after reports of nephrotic syndrome [265], membranous nephropathy [266], and acute or chronic renal failure [22,181,267] following argyria, but a causal relationship could not be indisputably defined. Still, it should be highlighted that the development of an antineutrophil cytoplasmic antibodies (ANCA) negative pauci-immune glomerulonephritis in a 47-year-old woman with a T-cell lymphoma has been reported, suggesting that the silver deposition in glomerular basement membranes, though seemingly innocuous, might be the trigger for leucocyte-mediated aggression in patients with a background of auto-immunity disorders [268].

Liver toxicity following silver deposition has also been suggested but, apart from transient hepatic enzyme elevation with no clinical significance, no evidence of pathological liver processes caused by silver accumulation has been found [5,55,182,223,269]. Indeed, most argyric patients, as well as individuals exposed to silver without clinical signs of argyria, present with corresponding laboratory parameters within reference values for healthy people [6,48,49,51,52,66,69,132,144,211,212,218,230,263]. Argyric patients might require special attention when radiotherapy is required after a report of a radiation dermatitis possibly explained/aggravated by the dermal silver deposition [215].

Lung fibrosis in silver finishers has also been reported [270,271]. Even though silver’s contribution to said outcome remains speculative, the inhalation of silver dust cannot be ruled out as a causative factor for pneumoconiosis.

It has also been considered that silver exposition might precipitate neurological and/or psychiatric events. There have been reports of argyric patients presenting with diverse neurological clinical pictures, ranging from acute seizures to neurodegenerative disorders or even peripheral neuropathy [32,128,183,195,217,222,272], but the potential causal relationship remains inconclusive as the cases reported are rare and do not allow extrapolation. Regarding argyria affecting psychiatric patients, the possible causal relationship seems to be the opposite, as delusional beliefs often are what leads to unmeasured silver consumption [195,211,214,224,273].

### 7.2. Medical Exams

To better characterize the suspected pigmentation and exclude the differential diagnosis, histopathological analysis of the affected tissue is particularly important [7]. After conventional tissue section processing and staining with hematoxylin and eosin, the dark deposits may be seen both in bright and dark field microscopy, with the latter being more sensitive as they appear refractile, arranged as described above in the pathophysiology section [7,55,84,87,133,139,213]. Ultrastructural analysis of skin biopsies, but also of the kidney and choroid plexus, allows visualization of electron-dense particles, typically round or ovoid in shape but sometimes more irregular, and with a greater axis ranging from 10 to 1000 nanometers [31,80,127,132,133,140]. Reflectance confocal microscopy has also been used, namely for LA work-up, as it can help quickly excluding melanocytic proliferation [133].

To establish an unequivocal diagnosis, an Energy-Dispersive X-ray Spectroscopy (EDXS) must be undertaken as this technique, whose apparatus is generally attached to electron microscopes, allows identification of the chemical elements found in the granules via analysis of their emitted energy spectrum, making it the gold-standard tool for argyria definite diagnosis [38,39,83,84,132,133,141,164]. While being non-invasive, it presents remarkable specificity and sensitivity (estimated limit of detection of 3 to 4 ppm) for silver, as well as for other chemical elements such as selenium and sulfur, and it might even allow monitoring of subclinical silver deposition in occupationally exposed workers [133,164,274]. Other documented auxiliary diagnostic methods are the neutron activating analysis, which allows determination of the silver content in the skin, hair, urine samples, or the whole body, but the advantages it can bring being routinely used in the clinical setting are scarce [126,275,276]. With resort to atomic absorption spectrometry and, more recently, inductively coupled plasma mass spectrometry [230], measurement of serum and urinary silver levels [9,54], as well as in feces, hair [28,35,263], kidney and liver wet tissue samples [180], might help to determine the body burden of silver, but these parameters have shown to be consistently reliable neither for risk assessment of argyria development nor for diagnosis of the condition [9,28,35,54,180,230,263].

Dermatoscopy is also often employed, particularly in the evaluation of LA, typically exhibiting a dark, dense, and homogenous pigmentation [82,83,87,89,133]. Enei et al. [71] showed that dermatoscopy might allow a peek into some detail of the histopathological pattern: dermal papillary silver seemingly appears as dots, perieccrine pigment as circumferences/ellipses, and the silver along the interpapillary dermis as linear structures. While it cannot diagnose argyria, it may provide helpful information in excluding differential diagnosis [65].

Slit-lamp biomicroscopy is the first step in the diagnostic approach of OA, revealing a particulate pattern in the cornea, but this technique, possibly complemented with confocal microscopy, should be employed for exposure monitoring, or if GA is suspected even without clinical evidence of OA, since cornea’s Descemet’s membrane displays silver deposits quite early following either systemic or local exposition, making slit-lamp examination a sensitive index for both GA and OA [102,117,118,120,157,162,199]. Other ophthalmological tests have also been reported, such as fluorescein angiography, optical coherence tomography, and electroretinogram, but their purpose so far has been mainly to find eye structural and functional changes related to argyria rather than establishing the diagnosis and with extrapolations limited due to the number and type of studies [149,156,157,158,218,222].

## 8. Treatment

Spontaneous argyria regression or any kind of intermittence cannot be expected, as the silver remains inert in the deposition sites indefinitely; in order words, the pigmentation is permanent without treatment [4,13,19,47,56,131,166]. At this point, the degree of sun exposition will determine the discoloration aggravation, with cessation of silver exposure being critical to avoid worsening of the aesthetical alteration, but seemingly futile in what concerns reverting it [5,34,39,178,223,225]. Therefore, the use of cosmetics can help to mask the discoloration, and sunscreens might be beneficial in preventing further pigmentation when sun exposure is unavoidable [7,36]. At the occupational level, periodic slit-lamp biomicroscopy and monitoring of silver aerial concentration have been suggested to ensure the employees’ safety [120,162,261]. Contrasting with these general findings, Wu et al. [121] reported a case of presumed OA following silver nitrate cautery of the palpebral conjunctiva with clinical and confocal microscopy regression with a follow-up of three years.

### 8.1. Treatment of Argyria

For a long time, there has not been an effective and safe treatment method for argyria [13,19,26,36,135,141]. Numerous approaches to remove the coloration are reported in the literature, but none with appreciable success: depigmenting creams, chelation therapy with different drugs such as 2,3-dimercapto-1-propanol (i.e., dimercaprol), 2,3-dimercapto-1-propanesulfonic acid (DMPS), sodium thiosulfate, potassium ferrocyanide, potassium iodide, ethylenediaminetetraacetic acid (EDTA), methenamine and D-penicillamine/*N*-acetyl-DL-penicillamine, hydroquinone and dermal abrasion all proved ineffective in removing silver deposits from the body [26,55,62,127,141,192,200,276,277,278,279]. Some of these chelating agents have been reported to lighten the pigmentation in small skin areas after intradermal injection, particularly sodium thiosulfate and potassium ferrocyanide, but results are inconsistent, and their application to larger areas would be quite incommodious and burdensome, with the risks associated far outweighing the unlikely benefits [19,55,131,178,245].

**Figure 6:**
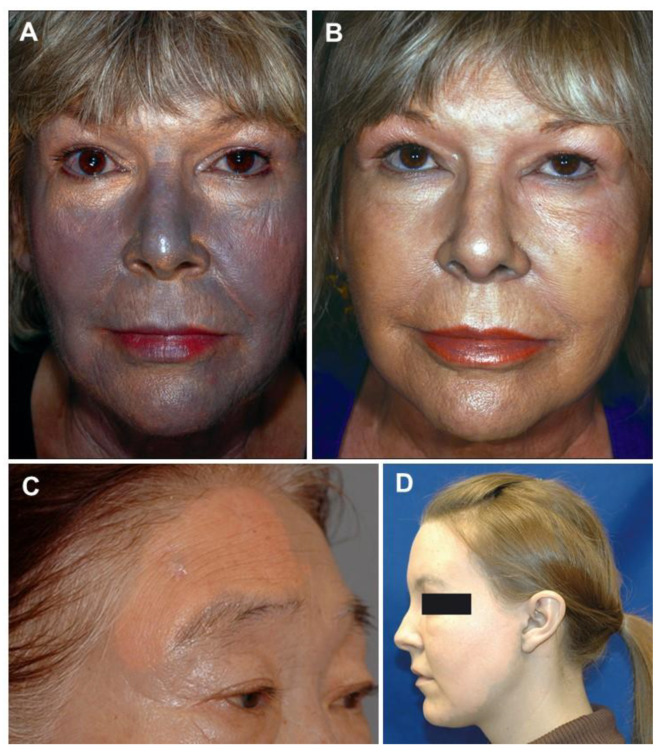
Laser treatment of argyria of the face and neck before and after a single pass of a Q-S 1064 nm Nd:YAG laser (**A**,**B**), notorious discoloration lightening of the right half of the forehead (**C**), and visible contrast between untreated (neck) and treated (face) areas (**D**). Reprinted from (**A**,**B**)—[63], (**C**)—[252] and (**D**)—[282].

In the last few years, cases of successful skin pigmentation resolution with conventional tattoo-removal laser techniques have been reported, offering insight in a promising treatment modality that might revolutionize argyria’s natural disease progression and the psychological burden. Both Q-Switched 1064 nanometers Neodymium-doped Yttrium Aluminum Garnet Laser (Q-S 1064 nm Nd:YAG laser) and Q-Switched Picosecond 755 nanometers Alexandrite Laser (Q-S P 755 nm Alexandrite Laser) have been reported to yield satisfactory results. The former, ranging from a fluence of 0.7 to 8 J/cm^2^, a pulse duration of 5 to 50 ns, a frequency of 5 to 10 Hz, and a spot size of 2 to 8 mm [139,219,223,252,280,281], produced immediate results, restoring the expected skin coloration for each subject in the targeted areas [51,63,142,282]. As with the latter, at least similar efficacy was obtained within reported values of 0.71 to 2.83 J/cm^2^ regarding fluence, 0.75 ns regarding pulse duration, 3 to 5.5 mm regarding spot size, and 10 Hz regarding frequency [74,216,219,220]. The persistence of restored skin coloration has been reported up to one year after treatment [142,223,252,282]. Otherwise, there has been one report of argyria pigmentation recurrence 11 months after Q-S 1064 nm Nd:YAG laser treatment, despite the patient stating discontinuation of silver exposure, use of facial and body sunscreen with a sun protection factor superior or equal to 30, and resort to physical sun-protective barriers [63,283]. Figure 6 evidence the effect of the treatment with Q-S 1064 nm Nd:YAG laser.

Both procedures provoke very intense pain [280], seemingly greater as fluence increases and more area is to be depigmented [282]. For this reason, technicians have resorted to varied anesthesia techniques, with general anesthesia being offered in some cases [51,142]. Beyond that and a transient inflammation of the skin with edema, erythema, and scaling [74,88], no other significant adverse effects or sequelae have been reported, with adequate healing of the treated areas [51,142,216,219,223,252,281,282].

The precise mechanism behind the disappearance of the pigmentation is not totally clear. It was initially postulated that the silver particles, once laser-targeted, would fragment, with their remnants being phagocytosed and removed from the skin via lymphatic uptake in a similar fashion to what occurs with conventional tattoos; this hypothesis is consistent with the decrease in the number of silver granules observed by light microscopy of post-treatment biopsies [223,252]. However, this mechanism would not be able to explain the macroscopical whitening of the skin obtained right after laser appliance, nor the fact that immediate post-treatment biopsies, taken from normal-appearing skin, revealed persistence of silver particles, which suggests laser treatment affects silver particles’ plasmon resonance, altering its emitted optical spectrum and allowing skin color restoration despite silver persistence [282,283]. It was speculated that Q-S 1064 nm Nd:YAG treatment might reverse the intradermal photoactivated reaction that silver particles go through after sunlight exposure, but their persistence in the skin allows argyria relapse if ultraviolet radiation reaccumulates [283]. Shao et al. [220] recently reported that after Q-S P 755 nm Alexandrite Laser, the silver particles, previously large enough to be seen by light microscopy with hematoxylin and eosin staining, were no longer able to be visualized by this means. Electron microscopy showed that the silver had not been removed from the skin but rather remained in similar locations, only now fragmented; particles went from approximately 25–100 nanometers to 4–15 nanometers, arranged in clusters. Two months after treatment with Q-S 1064 nm Nd:YAG laser, the persistence of perieccrine silver in macroscopically normal-appearing skin areas, documented with scanning electron microscopy, has been reported [282]. These data, along with Mock et al. [284] findings that heat treatment can modify silver nanoparticles’ geometrical shape, altering their emitted light spectrum wavelength, help put together the most robust explanation so far as to why clinical argyria provisional resolution, at least, can be obtained despite silver persistence in the skin and emphasizes the need not discard the possibility of argyria relapse even with silver exposure discontinuation.

### 8.2. Treatment of Amalgam Tattoo

The treatment of amalgam tattoos has also been an object of attention. The operator should irrigate the intervened area thoroughly to ensure all traces of silver-containing root canal sealer are properly cleaned up and removed to prevent the lesion onset, especially when the procedure requires soft tissue flap reflection [96]. After LA develops, if the lesion is cosmetically unacceptable, surgical excision and transplantation of oral mucosal tissue/free gingival grafting, as well as subepithelial connective tissue grafts, have been reported, with heterogenous results (even with LA resolution, the final aesthetic outcome might not be optimal) [92,206,207,285]. Regarding secondary prevention, periradicular surgery to remove the amalgam restorations and reduce the amount of dispersed silver particles before grafting the affected tissue has been reported [206].

### 8.3. Treatment of Argyrosis

Following acute angle-closure glaucoma, the use of Q-S Nd:YAG laser iridotomy in a patient with ocular argyrosis was reported, with each laser shot causing clearance of the argyrotic deposits anterior to the iridotomy site; the procedure was also performed in the contralateral eye as a preventive measure [151]. The cleared cornea areas remained unchanged for at least eight months, the follow-up period.

## 9. Forensic and Toxicological Aspects

Scarce *postmortem* evaluations of argyric patients have been reported [31,32,33,40,42,135]. A substantiated link between silver exposure and death was not found, with the patients often having many comorbidities or a clear non-related cause of death [21,31,32,33,40,42,135]. Still, these contributed to strengthening the notion that silver deposition can happen in several organs without leading to any clinically perceived consequences [31,33,40,42,135]. Autopsy findings after silver treatment of burn victims indicate the highest levels occur in the skin, gingiva, cornea, liver, and kidneys [15].

Silver and silver nanoparticles are relatively nontoxic, supporting their wide application [15,16,18,286]. Lesions of the kidneys and lungs and arteriosclerosis have been attributed to both industrial and medicinal exposures [15]. Deposition of silver in renal glomerular basement membranes after high-level exposure may produce hypertension and possible cardiac complications [15,287]. At lower exposure levels, more subtle effects of impaired endothelial function and inhibition of vascular endothelial growth factor action result in impaired angiogenesis and vasorelaxation [287,288]. The respiratory tract may be affected in severe cases of silver intoxication. Chronic bronchitis has also been reported to result from the medicinal use of colloidal silver. Large oral doses of silver nitrate or acetate may cause severe gastrointestinal irritation due to its caustic action [18]. After ingestion of 15 mL of silver nitrate solution, excruciating burning pain in the throat and nostrils was reported, but with the absence of any other symptoms or signs other than a whitish membrane on the oral mucosa, even after upper gastrointestinal endoscopy [289]. It has been reported that levels of AgNP administered i.v. at a dose above 20 mg/kg are toxic [290]. Silver has very limited genotoxic effects and is not considered a carcinogen.

## 10. Conclusions and Future Perspectives

A precise cut point for silver ingested after which GA development should be expected has not been determined, for which contributes to the rarity of this occurrence but also the unawareness of argyric patients regarding the silver products’ composition and their consumption magnitude [64,200,211,220,221].

**Figure 7:**
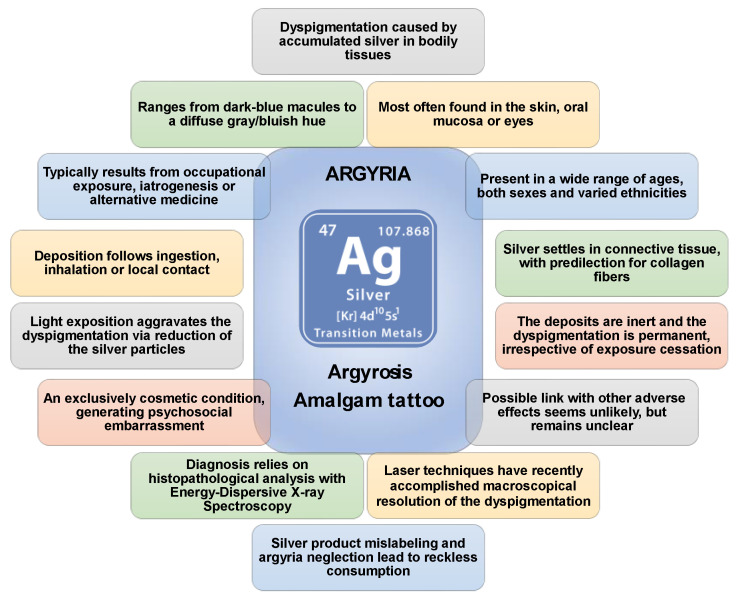
Major characteristics of different forms of argyria.

Argyria is the staining of tissues following silver accumulation [1,2,4]. It can be categorized as GA or LA, whether silver reaches the afflicted tissue(s) via the systemic bloodstream or local impregnation, respectively [44,70,72]. Furthermore, LA can be classified according to the site of deposition, with the most notorious types being cutaneous localized argyria, ocular argyrosis, and amalgam tattoos [2,97,112]. General major characteristics of argyria are resumed in Figure 7.

In the genesis of argyria development, several silver sources have been identified, which can be divided into three main categories: iatrogenic, occupational, and alternative medicine-related (Table 1). Currently, silver compounds in medicine have been refined, and indications for their use are restrained when compared to the past, being mainly related to the local application for asepsis insurance, wound cautery, and teeth restoration, avoiding systemic exposure [8]. Work conditions have also improved, with the advent of adequate ventilation and proper protective equipment, making the onset of argyria following occupational exposure a much rarer event [8,81]. These, along with hoodwinking marketing, mainly online, responsible for spreading misinformation about silver health benefits and neglecting its potential hazards, have caused an increase in the number of agyria case reports, particularly generalized argyria, with neither medical nor occupational genesis [20,66,89,200,244]. Except for light exposure, which plays an important role in aggravating the pigmentation, other factors besides silver exposition itself, contributing to argyria onset, are not known [48,79,130].

Argyric patients usually present with the absence of signs and symptoms related to argyria. What brings them forth is the aesthetical nuisance, which can lead to significant embarrassment and social withdrawal, even more after realizing that the pigmentation is permanent [9,132,143,223]. To diagnose argyria, physical examination and anamnesis alone can be very accurate, but maximum specificity is only achieved with EDXS of the stained tissue, as it allows ascertainment of silver present in the deposits, as well as quantification of other present nanoparticles [73,74,128,144,146].

Novel treatment modalities, with greater safety and effectivity than those ever achieved with previous techniques, now exist. Q-S 1064 nm Nd:YAG laser and Q-S P 755 nm Alexandrite Laser have shown potential to diametrically change argyria’s prognosis from permanent coloration to the restoration of previous complexion [216,219,223]. Future studies further looking into these techniques can help better clarify the mechanism of pigment resolution, optimize the results, and truly bring renewed hope to argyric patients.

## Figures and Tables

**Table 1:** Overview of major causes of argyria.

Treatment	Cause	References
Gastrointestinal conditions (ingestion of silver-containing colloids/pills)	Iatrogenic, systemic	[135,166,167,168,169,170,171,172,173,174]
Leukoplakia patch (topical application of silver nitrate)	Iatrogenic, topical	[175]
Epilepsy and other neuropsychiatric conditions (ingestion of silver-containing pills)	Iatrogenic, systemic	[40,131,176,177]
Alopecia (ingestion of silver-containing colloids)	Iatrogenic, systemic	[52]
Prophylaxis of gonococcal ophthalmia neonatorum (application of silver nitrate collyrium)	Iatrogenic, topical	[46,105,178]
Syphilis (topical application of silver arsphenamine)	Iatrogenic, topical	[126,131]
Wounds/ulcers/burns (topical application of silver sulfadiazine cream for asepsis, silver nitrate for chemical cautery/hemostasis, and/or use of silver-impregnated suture threads/surgical clips)	Iatrogenic, topical and/or systemic (if bloodstream is reached)	[70,84,85,105,128,141,179,180,181,182,183,184,185,186,187]
Strabismus surgery (application of silver nitrate collyrium and/or use of silver-impregnated suture threads/surgical clips)	Iatrogenic, topical	[160,161,188]
Trachoma (topical application of silver nitrate for chemical cautery)	Iatrogenic, topical	[121]
Conjunctivitis/eye soreness/epiphora (application of silver-containing collyrium)	Iatrogenic, topical	[114,153,154,189,190]
Pharyngitis/throat soreness (topical throat application of pulverized silver and/or ingestion of silver-containing tablets)	Iatrogenic, topical (pharyngeal) and/or systemic	[67,144,191,192,193,194]
Hematuria (instillation of the urinary tract with silver nitrate preparations)	Iatrogenic, topical	[105,106,107]
Smoking cessation (chewing/ingestion of silver-coated sugar particles and/or silver acetate lozenges/pills)	Iatrogenic, systemic	[57,126,195,196,197,198]
Varicose veins (injection of silver nitrate as sclerosant)	Iatrogenic, topical, and/or systemic	[130]
Intractable diplopia (use of silver nitrate-coated soft lenses)	Iatrogenic, topical	[199]
Antiseptic and astringent properties (application of silver-containing vasoconstricting nose drops)	Iatrogenic, topical, and/or systemic (silver is drained posteriorly and ingested)	[5,40,47,55,59,64,126,134,186,200,201,202,203,204,205]
Dental restoration (silver-containing filling material for endodontic procedures)	Iatrogenic, topical	[92,93,94,96,97,98,100,185,206,207,208,209]
Halitosis (silver-containing breath-freshening pills)	Non-medical, systemic	[8,210]
Belief in general health benefits/immune system boosting/alternative medicine (ingestion of silver-containing colloids, application of silver-containing nasal drops)	Non-conventional medicine, systemic	[1,6,9,20,22,34,38,44,45,49,50,51,58,62,66,68,69,125,211,212,213,214,215,216,217,218,219,220,221,222,223,224,225,226,227,228]
Skin-breaching trauma with silver-containing material	Accidental, topical	[145,229]
Antibiotic properties (use of silver-coated prosthetic implants)	Iatrogenic, topical, and/or systemic (if bloodstream is reached)	[90,230]
Photochemical industry	Occupational, topical (skin, eye, and intranasal deposition), and/or systemic (inhalation)	[112,152,231]
Occupational silver manipulation, silver soldering/silversmithing in jewelry/art crafting	Occupational, topical (skin and eye) and/or systemic (inhalation)	[54,77,80,119,122,150,163,232,233,234]
Eyelash tinting	Non-medical, topical	[115,235]
Acupuncture	Non-conventional medicine, topical	[8,89,98,139,140,236,237,238,239,240,241]
Silver earrings/piercings	Non-medical, topical	[2,71,72,74,88,137,138,165,242,243]
Silver-coated nuts and/or spices (areca and betel nut)	Non-medical, systemic (oral intake)	[7,91]

**Table 2:** Differential diagnosis for other argyria-mimicking pigmentations of skin and other tissues. GA, generalized argyria; LA, localized argyria.

Pathological Condition of Xenobiotic	Description	References
Hemochromatosis	Generalized skin hyperpigmentation (GA)	[40,56,64,69,126,134,167,178,245,246,247]
Lead poisoning	Blue line along the gingival margins at the base of the teeth (LA/amalgam tattoo)	[5,7,40,91]
Methemoglobinemia/sulfhemoglobinemia	Generalized skin brownish-blue to gray pigmentation (as in cyanosis) (GA)	[4,40,56,126,134,141,178,245,246,248]
Toxic melanodermatitis	Hyperpigmented skin lesions (LA, GA)	[132]
Minocycline	Blue staining of teeth or blue-gray skin lesions (GA)	[7,64,132,249]
Chlorpromazine/Phenothiazines	Slate gray-bluish skin discoloration in sun-exposed areas (GA)	[64,132,178,250]
Amiodarone	Slate gray-bluish skin discoloration in sun-exposed areas (GA)	[7,64,125,251]
Antimalarial agents	Blue-gray discolorations of the mouth and skin (LA, GA)	[55,79,132,246]
Clofazimine	Grayish skin plaques or generalized gray skin pigmentation (LA, GA)	[7,132]
Cyanosis/cyanotic heart disease	Generalized skin brownish-blue to gray pigmentation (GA)	[1,8,20,36,40,43,45,53,56,66,69,131,169,178,200,245,246,248,252,253,254,255,256]
Nevus	Flat or raised pigmented skin lesion (LA)	[2,71,73,76,82,133,137,164,229,241]
End-stage renal disease/uremia	Heterogenous skin lesions (LA, GA)	[36]
Melanoma of the skin/conjunctiva/oral mucosa	Pigmented lesion, usually asymmetric, with heterogenous color and time-evolving (LA/amalgam tattoo)	[23,82,92,95,96,100,101,119,133,159,178,188,240,246,257]
Chrysiasis	Slate-gray to blue skin pigmentation, especially in sun-exposed areas, nail pigmentation (LA, GA, azure lunula)	[36,64,79,113,178,237,246,258]
Iron salts	Brown to red skin lesions (LA)	[79]
Ochronosis	Bluish-black skin lesions (LA, GA)	[64,76,113,259,260]
Wilson’s disease	Generalized skin hyperpigmentation, Kayser-Fleischer rings (GA, OA)	[55,113]
Tobacco (chewing)	Brownish-black staining of the oral mucosa (LA/amalgam tattoo)	[91]
Chlorophyll (mouthwash)	Blackening of the tongue (LA/amalgam tattoo)	
Sodium perborate (mouthwash)	Blackening of the tongue (LA/amalgam tattoo)	
Ariboflavinosis	Diffuse bluish-purple discoloration of the buccal mucosa (GA)	
Peutz-Jeghers syndrome	Dark blue-brown hyperpigmented gingival macules (LA/amalgam tattoo)	
Addison’s disease	Generalized bronze-like skin pigmentation, diffuse pigmentation of gingiva, tongue, and buccal mucosa (GA)	[53,58,69,91,134,178,214,245]
Bismuthosis	Blue-black, sharply limited pigmentation of marginal gingivae, nail pigmentation (LA/amalgam tattoo, GA, azure lunula)	[36,79,91,134,178,246]
Mercurialism	Diffuse blue-gray to black gingival pigmentation, nail pigmentation (LA/amalgam tattoo, azure lunula)	[36,79,91,140,178]
Arseniasis	Generalized skin hyperpigmentation with hyperkeratosis (GA)	[5,7,79,91]
Accidental tattoo	Dark-blue/black macules/patterns (LA)	[133]

## Data Availability

Not applicable.
